# AcuTrials^®^: an online database of randomized controlled trials and systematic reviews of acupuncture

**DOI:** 10.1186/1472-6882-13-181

**Published:** 2013-07-19

**Authors:** Benjamin L Marx, Ryan Milley, Dara G Cantor, Deborah L Ackerman, Richard Hammerschlag

**Affiliations:** 1 Research Department, Oregon College of Oriental Medicine, 75 NW Couch St, Portland, OR 97209, USA

**Keywords:** Acupuncture, AcuTrials, CAM, Databases, Informatics, Evidence-based medicine

## Abstract

**Background:**

The growing quantity of Complementary and Alternative Medicine literature requires databases enabled with increasingly powerful search capabilities. To address this need in the area of acupuncture research, a bibliographic database of randomized controlled trials (RCTs) and systematic reviews called AcuTrials^®^ has been developed by the Oregon College of Oriental Medicine. AcuTrials^®^ introduces a comprehensive keyword thesaurus that categorizes details of treatment protocols and research design to an extent not currently available in MEDLINE or other databases.

**Description:**

AcuTrials^®^, which went live in January of 2010 and is updated monthly, currently contains over 1250 articles from more than 300 journals. Articles included are English language RCTs and systematic reviews that report on medical conditions in human subjects treated by needle acupuncture. Study details are indexed by 14 key domains, such as acupuncture style and needling protocol, to create an acupuncture-relevant, searchable keyword catalogue. Keywords follow the National Library of Medicine (NLM) MeSH terminology when possible, and new keywords were created in cases where no appropriate MeSH terms were available. The resulting keyword catalogue enables users to perform sensitive, targeted searches for particular aspects of acupuncture treatment and research design.

**Conclusions:**

AcuTrials^®^ provides an extensive and innovative keyword catalogue of acupuncture research, allowing users to efficiently navigate, locate and assess the evidence base in ways not currently possible with other databases. By providing a more powerful suite of search options, the AcuTrials^®^ database has the potential to enhance the accessibility and quality of acupuncture research.

## Background

Ideally, medical research should translate into improved patient care. For this translation to occur, health-care providers must keep informed of the current evidence base. However, identifying evidence to inform best-practice is an increasingly complex undertaking, a consequence of the profusion of medical literature and its dispersal over a broad range of journals and bibliographic databases. The sheer quantity of literature, varied in both publication site and indexing, presents a significant obstacle to an efficient and comprehensive assessment of current medical information. This obstacle is especially pronounced in the field of complementary and alternative medicine (CAM), where, among other difficulties, many CAM-relevant search terms either vary among databases or are non-existent [1-4].

Relative to biomedicine, the sources that catalog a comprehensive collection of CAM literature are considerably limited [5-7]. Some sources of CAM-specific literature include:

• The Natural Standard Database, an international research collaboration that aggregates and synthesizes evidence-based data on CAM [8].

• AMED, a bibliographic database produced by the Health Care Information Service of the British Library, which indexes a selection of journals in several subject areas including CAM [9].

• AltHealth Watch, a full-text database that indexes literature relevant to a wide range of CAM therapies [10].

• CAMbase, a bibliographical database containing more than 80,000 records from over 30 journals and periodicals on CAM [5].

A limited number of acupuncture-focused information services, such as Acubriefs (http://acubriefs.blogspot.com/) and the Acupuncture Research Resource Centre (http://www.acupunctureresearch.org.uk/), also provide access to some acupuncture literature, though locating a full and relevant collection of acupuncture research among these and other CAM resources presents particular challenges. Not only is acupuncture literature cataloged unevenly across databases [1], but the search-term indexing employed is inconsistent and often inadequate [2,11,12]. A survey of health sciences faculty affiliated with the University of California identified acupuncture among the major focus areas of literature searches they performed; yet, half the respondents perceived their CAM searches as being only partially successful [7]. Further confounding the issue, some acupuncture literature is published in print-only journals that are not currently indexed in any electronic database.

When searching for acupuncture related evidence, health professionals often rely on literature searches of PubMed [7]. However, many acupuncture publications are not yet indexed in this major bibliographic database [1,5,6]. Furthermore, developing an optimum PubMed search strategy for acupuncture research is problematic due to the paucity of specific Medical Subject Headings (MeSH) relevant to acupuncture [1,3]. Currently, the National Library of Medicine (NLM) Classification Outline (utilized on PubMed) lists only seven acupuncture-related MeSH terms: Acupuncture Therapy; Acupuncture Analgesia; Acupuncture, Ear; Electroacupuncture; Meridians; Acupuncture Points; and Moxibustion. The dearth of specific search terms for acupuncture research and treatment on PubMed often yields poor returns of relevant articles.

As a means of addressing these issues, the Research Department at the Oregon College of Oriental Medicine (OCOM) has developed AcuTrials^®^, an online database of acupuncture research. AcuTrials^®^ was developed with two main aims in mind: First, to provide a complete, web-based, searchable database of randomized controlled trials and systematic reviews of acupuncture; Second, to populate the database with a comprehensive, acupuncture-specific keyword list that will facilitate efficient, targeted searches of the acupuncture literature.

## Construction and content

AcuTrials^®^ (http://acutrials.ocom.edu/) was initially compiled from searches of five online databases dating back to their inception: PubMed, the Cochrane Library, AMED, AltHealth Watch, and CINAHL. Additionally, seven traditional Chinese medicinal journals that are not indexed online were hand searched [13]: Australian Journal of Acupuncture and Chinese Medicine, Focus on Alternative and Complementary Therapies, International Journal of Clinical Acupuncture, Journal of Acupuncture and Meridian Studies, Journal of Traditional Chinese Medicine, Medical Acupuncture, and the North American Journal of Oriental Medicine. Data entry began in earnest in 2004, and trademark registration was granted in 2008. AcuTrials^®^ is maintained by on-going, monthly searches of these same online databases and print-only journals. No experimental research on either humans or animals was performed in the creation or maintenance of the database.

Global searches are performed for each of the five online databases by the AcuTrials director who enters the search criteria “acupuncture or electroacupuncture” and filters results from the date of last search. All relevant hits are reviewed, and articles selected for inclusion are English language randomized controlled trials and systematic reviews reporting on medical conditions in human subjects treated by needle acupuncture. Examples of exclusion criteria are laser acupuncture, acupressure, and non-randomized controlled trials.

**Figure 1:**
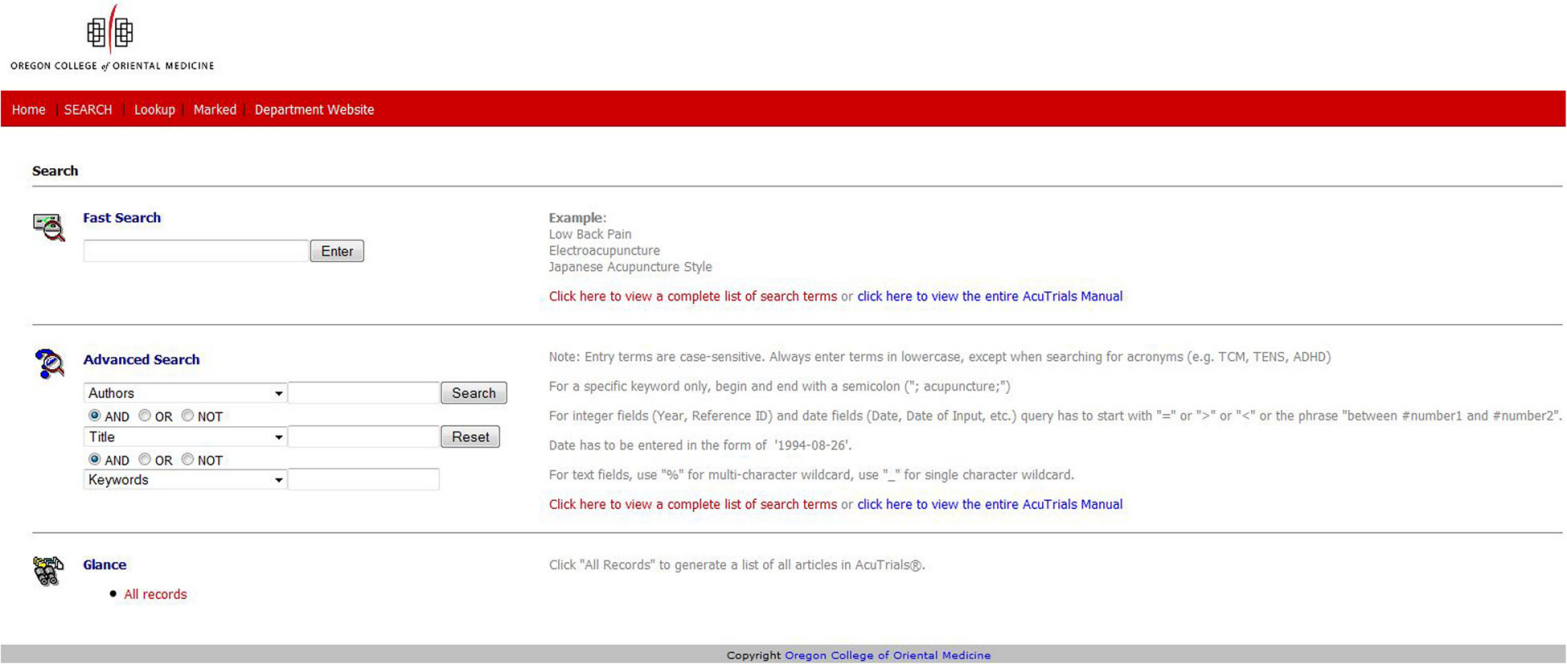
Search page.

**Figure 2:**
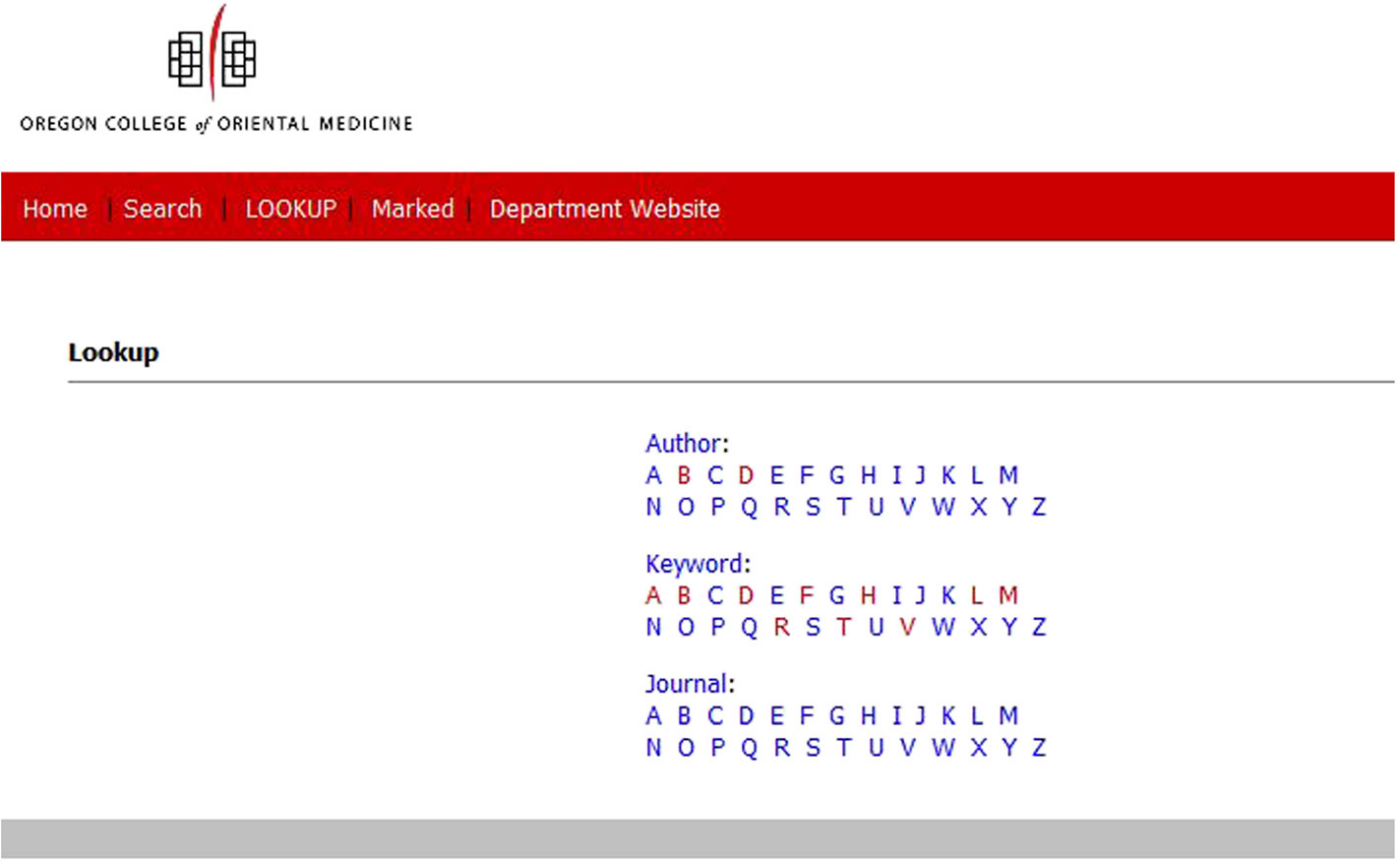
Hyperlinked Search Term Index.

As of July 1, 2013 AcuTrials^®^ contained over 1250 total articles, 1013 randomized controlled trials and 259 systematic reviews from more than 300 journals. On average, 7 new articles are added to the database each month. Since January of 2010, when the database was made available for beta-testing, over 3300 visitors from 28 countries have accessed information on the site. The database is open access, but requires each user to create a unique username and password. Search features allow users to rapidly retrieve relevant articles after entering simple keywords, or combine multiple keywords for more refined results (Figure 1). Users can also access articles by clicking on a hyperlinked index of search terms organized alphabetically by author, keyword, and journal (Figure 2).

### Utility

#### *Domains of data extraction*

Each article included in AcuTrials^®^ is entered through a systematic process to generate searchable keywords, resulting in greater breadth and specificity than is available in existing bibliographic databases. Two evaluators assess the full text of each article and compare extracted details to assure uniformity and control for quality. Discrepancies are resolved via discussion among evaluators and the AcuTrials director. The average time to enter an article into the database is approximately 15 minutes. For RCTs, data are extracted in 14 key domains, which cover most aspects of trial design and treatment (Table 1). For systematic reviews, data are extracted in only 3 of these domains (Disease Category, Condition Category, and Type of Study) due to the heterogeneity of trials included in a typical review. When possible, keyword cataloguing follows the NLM MeSH terminology. In cases where no appropriate MeSH term was available, a new term was created. The 14 areas of data extraction, and their associated keywords, are discussed in detail below.

**Table 1:** Domains of data extraction

1. Disease Category	8. Post Treatment Follow-up
2. Condition Category	9. Research Design
3. Type of Study	10. Control Group
4. Number of Subjects	11. Sham Control Group Details
5. Number of Treatments	12. Acupuncture Style
6. Duration of Treatment	13. Treatment Modalities
7. Frequency of Treatments	14. Needling Protocol

#### *Disease and condition categories*

Each article in AcuTrials^®^ is indexed with a keyword identifying the Disease Category—an overarching domain describing the disease under investigation, and the Condition Category—a term identifying the specific biomedical condition under investigation.

As an example, a search using the Disease Category term “Arthritis” will locate a broad range of trials reporting on numerous arthritic conditions; while a search with the Condition Category term “Osteoarthritis, Knee” will generate a select list of trials specifically regarding this condition (see Table 2). Currently, there are 39 Disease Category terms and over 300 Condition Category terms indexed in AcuTrials^®^. For a complete list of these terms visit http://www.ocom.edu/images/pdf/adc.pdf or connect to the term list via the AcuTrials^®^ website, http://acutrials.ocom.edu/.

**Table 2:** Disease and condition categories

**Disease category**	**Associated condition categories**
Arthritis	Arthritis; Arthritis, Rheumatoid; Osteoarthritis; Osteoarthritis, Hip; Osteoarthritis, Knee; Osteoarthritis, Spine

#### *Study details*

AcuTrials^®^ indexes study data into six domains considered particularly important in acupuncture trials: 1) Type of study (i.e. RCT or Systematic Review), 2) Number of subjects, 3) Number of treatments, 4) Duration of treatment 5) Frequency of treatments, and 6) Length of post-treatment follow-up.

These data can be used by clinicians for quickly elucidating the number and frequency of treatments reported when developing treatment plans for patients. Researchers and policy makers can use these data to efficiently sort through trials of various subject sizes.

#### *Research design*

Research designs vary widely in acupuncture trials. The structure of a trial, including such variables as the number of control/comparison groups, and whether a sham control is included, can influence study outcomes and interpretation. No MeSH terms currently exist to reflect the diversity of commonly employed research designs in acupuncture trials. AcuTrials^®^ contains twelve unique search terms developed to address this deficit (Table 3). Each RCT in AcuTrials^®^ is indexed with 1 of these 12 Research Design terms.

**Table 3:** Research design

1. Acu *Versus* Active Control	7. Acu *Versus* > 1 Control
2. Acu *Versus* Acu	8. Acu *Versus* Usual Care
3. Acu *Versus* Attention Control	8a. Acu + Usual Care *Versus* Sham + Usual Care
4. Acu *Versus* No Treatment	8b. Acu + Usual Care *Versus* Usual Care
5. Acu *Versus* Sham	8c. Acu *Versus* Sham + Usual Care
6. Acu *Versus* Wait List	8d. Acu + Usual Care *Versus* > 1 Control

These distinctions could, for example, allow clinicians to limit a search to RCTs comparing two different types of acupuncture treatments for a given condition, e.g. *Acu Versus Acu AND neck pain*. For acupuncture researchers, a search of these indexed keywords can quickly generate a list of all trials employing a particular design. For example, *Acu Versus Sham* will locate all trials with designs comparing a real acupuncture treatment against a sham acupuncture treatment. As a further example, policy-makers, as well as patients, may be interested in searching for trials comparing acupuncture treatment to an accepted biomedical treatment, such as a pharmaceutical or physical therapy (also called comparative effectiveness research). The keyword *Acu Versus Usual Care*, which is further refined into several sub-categories (Table 3, items 8a-8d), can accommodate this.

#### *Control group*

**Figure 3:**
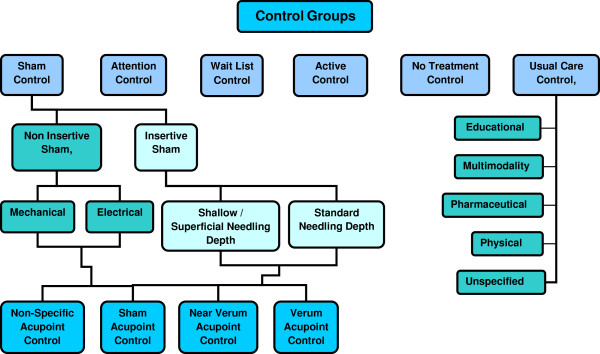
Control Group Keywords.

Many types of control groups have been employed in RCTs of acupuncture. Some trials compare acupuncture treatment to an accepted biomedical treatment, while others compare treatment to a sham needling or placebo treatment. To enable refined searches among the range of control groups, six primary keywords were created, along with further keywords to more specifically describe the Sham and Usual Care control groups (Figure 3).

1. *Sham Control:* A control treatment designed to mimic true needling but have no intended therapeutic action (e.g. toothpick in guide tube, sham needling device, superficial needling).

2. *Attention Control:* No treatment other than attention from a clinician during a visit.

3. *Waitlist Control:* Treatment delayed; subjects initially serve as No Treatment Controls.

4. *CAM Control:* An active intervention not considered Usual Care or a Sham (e.g. acupuncture, massage, herbs, chiropractic, applied relaxation, Reiki, Qigong, aromatherapy, bee venom, naturopathy, etc)

5. *No Treatment Control*: No other treatment.

6. *Usual Care Control:* A typical, accepted biomedical treatment. Five terms (educational, multimodality, pharmaceutical, physical, and unspecified) are used to further qualify the type of biomedical care.

#### *Sham control group details*

Sham control procedures utilized in clinical trials of acupuncture can be categorized as one of two distinct types: insertive or non-insertive. Within each of these categories, further refinement is possible based on the location of the sham points. To capture these distinctions, several additional keywords that reflect the breadth of acupuncture sham controls are indexed:

1. *Insertive Sham*: The sham control penetrates the skin. ‘Shallow/Superficial’ and ‘Standard’ are two further keywords used to describe the depth of insertion.

2. *Non-Insertive Sham*: The sham control does not penetrate the skin. ‘Mechanical’ and ‘Electrical’ are two further terms used when appropriate.

3. *Verum Acupoint Control*: The control point locations are the same as the treatment point locations.

4. *Near Verum Acupoint Control*: The control points are non-acupoint sites described as in the vicinity of actual acupoints, (e.g. 2 mm away from LI-4).

5. *Sham Acupoint Control*: The control points are non-acupoint sites away from all recognized verum acupoints (e.g. center of the knee cap, between BL and GB meridians away from all know acupoints).

6. *Non-Specific Acupoint Control*: The control points are real acupoints not known to be effective for the condition under investigation.

This level of specificity can be especially useful for researchers interested in exploring the relationships between control type and outcomes. For example, researchers can investigate if trials employing non-insertive shams or insertive shams are more likely to produce differences compared to verum needling.

#### *Acupuncture style*

Styles of acupuncture vary widely due to cultural influence, geographic location, and differences in underlying theory. For example, Japanese meridian therapists generally employ superficial needling techniques (1-5 mm depth) with short needle retention time (1-5 minutes), whereas practitioners of Traditional Chinese Medicine (TCM) generally utilize deeper insertions (10-50 mm depth) with longer needle retention times (20-30 min). No MeSH terms currently exist to reflect the variety of styles employed in acupuncture trials. To reflect this diversity, each study is indexed with one of fourteen keywords created to describe the acupuncture style utilized in the trial (Table 4).

**Table 4:** Acupuncture styles

1. 5-Phase Acupuncture Style	8. Korean Hand Acupuncture Style
2. Ashi Acupuncture Style	9. NADA Protocol Acupuncture Style
3. Dry Needling, with Acupuncture Needle	10. Nogier Acupuncture Style
4. Dry Needling, with Non-Acupuncture Needle	11. Other Acupuncture Style
5. French Meridian Acupuncture Style	12. TCM Acupuncture Style
6. Japanese Acupuncture Style	13. Trigger Point Acupuncture Style
7. Korean Acupuncture Style	14. Unspecified Acupuncture Style

#### *Treatment modalities*

Acupuncture is one treatment modality within a system of care often called Traditional East Asian Medicine (TEAM). Other modalities include herbal medicine, moxibustion, cupping, and massage. Some studies of acupuncture reflect a whole-systems approach by coupling acupuncture treatment with one or more TEAM modalities, many of which have not been categorized with MeSH terms. In cases where a trial employed an auxiliary intervention, a keyword was created to describe this additional component of treatment (Table 5). For example, a trial that includes herbal medicine for the treatment of headaches is indexed with the keyword ‘Herbal Formula.’

**Table 5:** Treatment modalities

1. Abdominal Acupuncture	13. Herbal Acupoint Injection
2. Acupressure	14. Intradermal Needles
3. Auricular Acupuncture	15. Laser Acupuncture
4. Auricular Electroacupuncture	16. Moxa, Direct
5. Bloodletting	17. Moxa, Indirect
6. Cupping	18. Plum Blossom
7. Ear Seeds	19. Press Tacks
8. Electroacupuncture	20. Qi Gong, External
9. Floating Acupuncture	21. Qi Gong, Internal
10. Hand Acupuncture	22. Scalp Acupuncture
11. Herbal Formula	23. Scalp Electroacupuncture
12. Herbal IV	24. Tuina

Additionally, in cases where a variant of standard body acupuncture was used in the study, a keyword was included to describe this variation in treatment (Table 5). For example, a trial that utilizes ear acupuncture for the treatment of anxiety is indexed with the keyword ‘Auricular Acupuncture’ and ‘Anxiety.’ Clinicians interested in enhancing an evidence-informed practice [14,15] can use these keywords to locate trials showing how the addition of an auxiliary modality impacted treatment outcomes, e.g. headache AND herbal formula. As another example, researchers conducting a systematic review can use these keywords to quickly generate a list of trials employing a specific treatment for a particular condition, e.g. stroke AND scalp acupuncture.

#### *Needling protocol*

Acupuncture trials differ in the way acupuncture points are selected and used. Referred to as the ‘Needling Protocol’ in AcuTrials^®^, some protocols limit all treatments to the exact same set of acupuncture points, while others allow for changes in acupoint selection from patient to patient, or treatment to treatment. To enable refined searches among the range of needling protocols, six keywords were created:

1. *Manualized Acupuncture Protocol:* The theoretical framework, diagnosis algorithm, and needling protocol is pre-determined and clearly reported.

2. *Individualized Acupuncture Protocol:* The patients are treated individually, points are uniquely grouped and chosen, and no restrictions on point selection are described.

3. *Semi-individualized Acupuncture Protocol*: A core set (or sets) of points and principles are used on all patients, with modifications allowed.

4. *Fixed Acupuncture Protocol:* All patients receive the same acupuncture points.

5. *Ashi Point Acupuncture Protocol:* The needle placement is determined by palpation of tender points in the muscle and “Ashi” is mentioned in the study methods.

6. *Trigger Point and Other Acupuncture Protocol:* The term “Trigger Point” is mentioned as a point selection method, or this keyword is used for an alternatively described point selection protocol not encompassed by the other terms.

Needling Protocol terms can be used by clinicians and researchers interested in retrieving studies utilizing a specific acupuncture protocol for a condition, e.g. fixed acupuncture protocol AND hot flashes, or for quickly locating studies employing a more real-world approach (to better achieve model-fit validity) such as trials employing a ‘Manualized’ or ‘Individualized’ Acupuncture Protocol.

## Discussion

AcuTrials^®^ addresses deficits in existing acupuncture literature databases in two primary ways. It provides a universally accessible (i.e. web-based), comprehensive database of acupuncture RCTs and systematic reviews, and it introduces a searchable, acupuncture-specific keyword list to enable users to perform sensitive, targeted searches of the research.

The specificity of the keyword indexing in AcuTrials^®^ allows for sorting and sifting of trials in ways previously not possible. While some CAM databases provide broad brush-stroke syntheses of trial outcomes, the greater level of indexing in AcuTrials^®^ allows for more nuanced searches of the acupuncture research literature. Clinically, providers can efficiently identify condition-specific treatment protocols when they wish to integrate current research findings into practice. Often defined as an ‘evidence-informed practice,’[14,15] this infusion of current research findings into the clinical domain is often under-utilized by acupuncture practitioners [16,17]. An evidence-informed approach is increasingly championed by policymakers as an essential component in modern clinical decision-making. Furthermore, because the database allows for rapid access to comparative effectiveness trials, primary care physicians may find this an efficient tool when making recommendations to the increasing number of patients seeking acupuncture care [18,19]. Likewise, acupuncture and biomedical practitioners have rapid access to trials that utilize acupuncture adjunctive to bio-medical care. Timely access to these trials provides best evidence for conditions in which acupuncture should be utilized as an adjunctive component of care. For example, in cases of physical therapy and pharmaceutical management of osteoarthritis of the knee, the addition of acupuncture has demonstrated an enhanced effect [20,21]. Streamlining the process of information acquisition and assessment for the evidence-informed practitioner makes AcuTrials^®^ a useful point-of-care tool.

Many of the searchable keyword domains in AcuTrials^®^ mirror the 2010 revised Standards for Reporting Interventions in Clinical Trials of Acupuncture (STRICTA) [22] checklist of essential reporting items, allowing researchers to perform more efficient, targeted literature searches based on these important components. For example, AcuTrials^®^ was utilized to expand search capabilities for a systematic evaluation of acupuncture RCTs conducted between 1997 and 2007 [23]. The evaluation of 333 trials reported during this 10-year period indicated improving reporting quality, and more importantly, showed that the overall quality of acupuncture research is on par with research of general medical interventions.

The level of specificity in the keyword indexing of AcuTrials^®^ may also make it possible to pose broader questions about acupuncture research than was previously possible. For example: What impact do individual needling protocols, as compared to fixed needling protocols, have on trial outcomes? What is the impact of treatment frequency or duration on outcomes? Does the addition of auxiliary TEAM modalities improve outcomes, and if so, which modalities, and for which conditions? The database can also be used to assist in investigating methodological design issues currently debated in acupuncture research, such as: What is the place of sham or placebo controls in acupuncture trials, and in particular, which type of sham is most appropriate? Answering such nuanced questions is feasible, as all the RCTs in AcuTrials^®^ are indexed with data and search terms detailing these specific aspects of the trials.

In its current form, the database has several limitations due to insufficient resources. First, RCTs and systematic reviews are the only trial designs indexed at present. Other forms of research such as non-randomized controlled trials, outcomes research, animal studies, mechanistic explorations, and case studies are excluded. These types of evidence are a key component of a comprehensive evidence base, and of foundational importance from both a clinical and research standpoint. Also missing are primary trials of other TEAM modalities (e.g. Chinese herbs, acupressure, moxibustion, Tuina), and non-English language publications. It is often argued that to be comprehensive, the search methods for systematic reviews should include non English language literature [24,25]. Given that a significant portion of TEAM trials are likely to be published in Chinese language journals [26], the scope of AcuTrials would be considerably expanded with the addition of trials in languages other than English.

Lastly, AcuTrials^®^ is limited by its current software platform, which does not allow customization of the user interface. With more customizable software, AcuTrials^®^ could enhance the user experience in a number of ways, including: 1) A more sensitive advanced search function, making it easier for users to take advantage of the full range of search terms; 2) A “smarter” keyword thesaurus, which could recognize lay terms when entered and link to the appropriate keyword; 3) A hyperlinked keyword tree that would allow users to visualize the relationships between conditions and quickly link to relevant trials.

## Conclusions

As an online database of acupuncture RCTs and systematic reviews utilizing an innovative keyword indexing system, AcuTrials^®^ is a valuable resource for the CAM community. The database provides an extensive keyword catalogue for acupuncture research, allowing users to efficiently navigate and locate literature in ways not currently possible with other databases. The AcuTrials^®^ database, with its unique set of features, will help to advance both the accessibility and quality of acupuncture research.

## Availability and requirements

The database is accessible at http://acutrials.ocom.edu/. The database is open access, but requires each user to create a unique username and password.

## Abbreviations

CAM: Complementary and Alternative Medicine; MeSH: Medical Subject Heading; NLM: National Library of Medicine; OCOM: Oregon College of Oriental Medicine; RCT: Randomized Controlled Trial; TCM: Traditional Chinese Medicine; TEAM: Traditional East Asian Medicine.

## Competing interests

The authors declare that they have no competing interests.

## Authors’ contributions

BLM managed the project, participated in the design of the database, migrated the data to its current software, and helped draft the manuscript. RM conceived of and built the database from inception into its early phases, established the keyword catalogue, guided and directed the project throughout, and helped draft the manuscript. DGC helped to enter content into the database, performed quality control and database improvements, and helped draft the manuscript. DLA supported the project and helped draft the manuscript. RH participated in the early design and concept of the database, supported the growth of the project, helped to refine the keyword catalogue, and helped draft the manuscript. All authors read and approved the final manuscript.

## Pre-publication history

The pre-publication history for this paper can be accessed here:

http://www.biomedcentral.com/1472-6882/13/181/prepub
